# Polymorphism rs2073618 of the *TNFRSF11B* (*OPG*) Gene and Bone Mineral Density in Mexican Women with Rheumatoid Arthritis

**DOI:** 10.1155/2017/7680434

**Published:** 2017-07-05

**Authors:** C. A. Nava-Valdivia, A. M. Saldaña-Cruz, E. G. Corona-Sanchez, J. D. Murillo-Vazquez, M. C. Moran-Moguel, M. Salazar-Paramo, E. E. Perez-Guerrero, M. L. Vazquez-Villegas, D. Bonilla-Lara, A. D. Rocha-Muñoz, B. T. Martín-Marquez, F. Sandoval-Garcia, E. A. Martínez-García, N. S. Fajardo-Robledo, J. M. Ponce-Guarneros, M. Ramirez-Villafaña, M. F. Alcaraz-Lopez, L. Gonzalez-Lopez, J. I. Gamez-Nava

**Affiliations:** ^1^Unidad Biomédica 02, Unidad Médica de Alta Especialidad (UMAE), Hospital de Especialidades, Centro Médico Nacional de Occidente (CMNO), Instituto Mexicano del Seguro Social (IMSS), 44340 Guadalajara, JAL, Mexico; ^2^Doctorado en Farmacología, Centro Universitario de Ciencias de la Salud (CUCS), Universidad de Guadalajara (U. de G.), 44340 Guadalajara, JAL, Mexico; ^3^División de Ciencias de la Salud, Departamento de Ciencias Biomédicas, Centro Universitario de Tonalá (CUTonalá), U. de G., 48525 Tonalá, JAL, Mexico; ^4^Instituto de Investigación en Reumatología y del Sistema Músculo Esquelético, CUCS, U. de G., 44340 Guadalajara, JAL, Mexico; ^5^UDG-CA-703, Grupo de Investigación en Inmunología y Reumatología, CUCS, U. de G., 44340 Guadalajara, JAL, Mexico; ^6^Departamento de Biología Molecular y Genómica, CUCS, U. de G., 44340 Guadalajara, JAL, Mexico; ^7^División de Investigación, UMAE, Hospital de Especialidades, CMNO, IMSS, 44349 Guadalajara, JAL, Mexico; ^8^Departamento de Farmacobiología, Centro Universitario de Ciencias Exactas e Ingeniería (CUCEI), U. de G., 44430 Guadalajara, JAL, Mexico; ^9^Departamento de Epidemiología, Unidad Médica Familiar 4 y 8, IMSS, 44220 Guadalajara, JAL, Mexico; ^10^Departamento de Salud Pública, CUCS, U. de G., 44340 Guadalajara, JAL, Mexico; ^11^Departamento de Medicina Interna-Reumatología, Hospital General Regional 110, IMSS, 44710 Guadalajara, JAL, Mexico; ^12^División de Ciencias de la salud, Departamento Salud-Enfermedad como Proceso Individual, CUTonalá, U. de G., 48525 Tonalá, JAL, Mexico; ^13^UDG CA-701 Grupo de Investigación Inmunometabolismo en Enfermedades Emergentes (GIIEE), CUCS, U. de G., 44340 Guadalajara, JAL, Mexico; ^14^Laboratorio de Investigación y Desarrollo Farmacéutico, CUCEI, U. de G., 44430 Guadalajara, JAL, Mexico; ^15^Unidad Medica Familiar 97, IMSS, 46470 Magdalena, JAL, Mexico; ^16^Programa de Doctorado en Ciencias Médicas, Universidad de Colima, 28040 Colima, COL, Mexico; ^17^Departamento de Medicina Interna-Reumatología, Hospital General de Zona 45, IMSS, 44860 Guadalajara, JAL, Mexico

## Abstract

Osteoporosis (OP) is highly prevalent in rheumatoid arthritis (RA) and is influenced by genetic factors. Single-nucleotide polymorphism (SNP) rs2073618 in the *TNFRSF11B* osteoprotegerin (*OPG*) gene has been related to postmenopausal OP although, to date, no information has been described concerning whether this polymorphism is implied in abnormalities of bone mineral density (BMD) in RA. We evaluated, in a case-control study performed in Mexican-Mestizo women with RA, whether SNP rs2073618 in the *TNFRSF11B* gene is associated with a decrease in BMD. RA patients were classified as follows: (1) low BMD and (2) normal BMD. All patients were genotyped for the *rs2073618* polymorphism by PCR-RFLP. The frequency of low BMD was 74.4%. Higher age was observed in RA with low BMD versus normal BMD (62 and 54 years, resp.; *p* < 0.001). Worse functioning and lower BMI were observed in RA with low BMD (*p* = 0.003 and *p* = 0.002, resp.). We found similar genotype frequencies in RA with low BMD versus RA with normal BMD (GG genotype 71% versus 64.4%, GC 26% versus 33%, and CC 3% versus 2.2%, resp.; *p* = 0.6). We concluded that in Mexican-Mestizo female patients with RA, the rs2073618 polymorphism of the *TNRFS11B* gene is not associated with low BMD.

## 1. Introduction

Rheumatoid arthritis (RA) is a systemic autoimmune disease characterized by a chronic inflammation of the synovial joints, resulting in bone erosions, joint deformities, subluxations, and sequelae that might lead to functional disability [1]. Osteoporosis (OP) is observed in 31% in Mexican women with RA [2]. OP constitutes the main risk factor for the development of osteoporotic fractures observed in around 4–23% of patients with RA [3–5]. Additionally, the risk of OP in RA is 2-fold higher compared with that in the general population [6]. Because OP comprises a multifactorial complication in RA, the genetic characteristics comprise one of the main factors implicated in the development of OP.

Osteoprotegerin (OPG) is a tumor necrosis factor receptor superfamily member (TNFRSF) that exerts protective properties against OP. Under normal conditions, OPG inhibits the maturation and activation of osteoclasts, acting as a protector for OP [7, 8]. Additionally, OPG shares a similar molecular structure with receptor nuclear factor kappa B (RANK), which is a molecule required for the linking of receptor nuclear factor kappa B ligand (RANKL); the latter molecule is implicated in the development of osteoclastogenesis with subsequent increased bone resorption, leading to OP [9, 10]. OPG acts on linking with RANKL, avoiding the formation of the RANK-RANKL complex, leading to osteoclast apoptosis [8].

OPG is encoded in humans by the *TNFRSF11B* gene that is located at 8q24.12 and that contains several polymorphisms, including the rs2073618 G>C transversion in exon 1, resulting in changes from Lys3Asn, the third amino acid of the signal peptide from lysine (AAG), into asparagine (AAC) [11, 12].

Several studies disagree concerning the role of the polymorphism in rs2073618 in postmenopausal OP, whereas some of these have suggested that it might constitute a genetic factor for OP [13]; others have found no relationship with postmenopausal OP [12, 14, 15]. However, the role of this polymorphism in the *TNFRSF11B* gene has not, to our knowledge, been evaluated in RA-associated OP.

Therefore, the aim of the present study was to assess whether there is an association among single-nucleotide polymorphisms (SNP), the rs2073618 *TNFRSF11B* (*OPG*) gene, and OP in Mexican-Mestizo women with RA.

## 2. Materials and Methods

### 2.1. Study Design

This study is a case-control study.

### 2.2. Clinical Setting

This study included patients with RA referred from two secondary-care hospitals (Hospital General Regional 110 [HGR110] and Hospital General de Zona 45 [HGZ45] of the Mexican Institute for Social Security (IMSS)) for performing a bone mineral density (BMD) scan in the bone mineral densitometry area at a tertiary-care center (UMAE, Hospital de Especialidades, Centro Médico Nacional de Occidente [CMNO]) in Guadalajara, Mexico.

### 2.3. Subjects

One hundred seventy-six patients were included in the study complying with the following inclusion criteria: (a) female, (b) >40 years of age, (c) Mexican Mestizos defined according to the Mexican National Institute of Anthropology and History (INAH) as “individuals who were born in Mexico, of the 3rd generation including their own and who were descendants of the original autochthonous inhabitants of the region and individuals who were mainly Spaniards” [16], and (d) those who met the American College of Rheumatology (ACR) 1987 criteria for RA. Patients were excluded if they had overlapping syndrome or if they were receiving antiresorptive treatment, such as prednisone ≥ 15 mg/day, or other therapies with biologic disease-modifying antirheumatic drugs (bDMARDs). We also excluded patients with chronic infectious diseases including hepatitis B and C or HIV infections and patients with chronic renal failure, transaminasemia (>2-fold normal values), or cancer.

### 2.4. Clinical Assessments

All patients were assessed for clinical and sociodemographic characteristics. Disease activity was assessed using the 28-joint Disease Activity Score (DAS28), and functioning was evaluated with the Health Assessment Questionnaire-disability index (HAQ-Di). Rheumatoid factor (RF) and C-reactive protein (CRP) were quantified in serum using nephelometry, and erythrocyte sedimentation rate (ESR) was determined utilizing the Wintrobe method.

### 2.5. Bone Mineral Density (BMD) Measurements

BMD (g/cm^2^) of the lumbar spine (L1–L4) and total hip was determined using dual-energy X-ray absorptiometry (DXA) with a Lunar Prodigy Advance densitometer (GE Medical Systems Lunar ver. 8.8 software; GE Medical Systems, Madison, WI, USA). According to the *T*-score of the BMD results, we classified the latter into two groups employing the 1994 World Health Organization (WHO) criteria as follows: group 1—low bone density if BMD in the lumbar spine or total hip had a *T*-score of ≤1.0 standard deviation (SD) (cases), and group 2—normal central BMD if the *T*-score of these two regions was >1.0 SD (controls).

For obtaining Hardy-Weinberg equilibrium, we used a group of 80 healthy blood donors obtained from the previously mentioned hospitals.

### 2.6. Genotyping

Genomic DNA from 256 subjects was extracted from peripheral blood leukocyte samples using the modified Miller technique [17]. The genotype was screened by an approach based on polymerase chain reaction-restriction fragment length polymorphisms (PCR-RFLPs). The 147 bp PCR product was incubated at 55°C with 5 U of *SmlI* restriction endonuclease as described by Langdahl et al. [15]. The resulting fragments were analyzed by electrophoresis in 6% followed by silver staining. The resulting genotypes for polymorphism were classified into one of the following three categories: nonexcisable homozygote (GG), excisable homozygote (CC), and heterozygote (CG).

### 2.7. Quantification of Serum OPG

Serum OPG levels were quantified by the enzyme-linked immunosorbent assay (ELISA) using a commercial human monoclonal OPG kit (RayBioTech Inc., Norcross, GA, USA). Characteristics of the OPG kit include a 1.0–900 pg/mL detection range and minimal detectable doses of 1.0 pg/mL.

### 2.8. Other Laboratory Tests

We quantified the levels of rheumatoid factor (RF) (IU/mL) and C-reactive protein (CRP) (mg/mL) by nephelometry. We also quantified erythrocyte sedimentation rate (ESR) (mm/h). We also included the quantification titers of antibodies directed against citrullinated proteins (ACPAs): second-generation anticyclic citrullinated peptide/protein antibodies of the second generation (anti-CCP2) and antimutated citrullinated vimentin antibodies (anti-MCV). We were able to determine one of these ACPAs in 115 patients. We determined anti-CCP2 in 112 patients and anti-MCV in 109 patients. But, both ACPAs (anti-CCP2 and anti-MCV) were determined only in 106 patients.

### 2.9. Statistical Analysis

Qualitative variables were expressed as frequencies (%), while quantitative variables were indicated as means ± standard deviation (SD). We identified genotype frequencies by direct counting. Allele frequencies were determined by counting from the observed genotype frequencies. Comparisons in means between the two groups were computed using the independent sample Student *t*-test, and comparisons among three subgroups, including (a) RA + OP, (b) RA + osteopenia, and (c) RA with normal BMD, were performed with one-way ANOVA. Dunnett correction was used as the post hoc test in case of statistical significance in the ANOVA. Comparisons between proportions were carried out using the chi-square test (or the Fisher exact test if required). Odds ratios (OR) and their 95% confidence intervals (95% CI) were calculated.

Hardy-Weinberg equilibrium in healthy subjects was determined by comparing the observed and expected data employing the chi-square test. Comparisons in OPG serum levels between each genotype of the rs2073618 polymorphism genotypes were performed by the Kruskal-Wallis test. In the present study, we employed, for comparison, the genotype frequencies of the recessive model GC + CC versus the frequencies of the GG genotype in each group.

A *p* value was considered significant at the *p* ≤ 0.05 level. To identify the variables associated with low BMD, we performed a multivariate logistic regression analysis, where low BMD was the dependent variable. We included, as covariates, variables such as age, BMI, positive ACPAs, glucocorticoid dose, and genotypes. The covariates included in the final model had *p* < 0.20 on univariate comparison or variables with biological plausibility using the stepwise method to identify associated variables, excluded potential confounders. Data were analyzed with the SPSS ver. 23.0 statistical software program (SPSS Inc., Chicago, IL, USA), and OR and their 95% CI were obtained utilizing EPI-INFO ver. 7.2 software (Epi Info™; Atlanta, GA, USA).

### 2.10. Ethics

The study protocol was approved by the Research and Ethics Board of the Hospital (R2007-1301-1). All study participants voluntarily provided written informed consent. All procedures in the protocol were performed according to the Declaration of Helsinki guidelines.

## 3. Results

We assessed 176 women with RA. Low BMD was observed in 74.4% of these patients, whereas OP was detected in 46.6%. Genotype distributions of this SNP in the group of 80 healthy subjects were consistent with the Hardy-Weinberg equilibrium (*p* > 0.64).

Table 1 presents two different comparisons. The first comprises the comparison between RA with low BMD (group 1; *n* = 131) and RA with normal bone density (group 2; *n* = 45). In the first comparison, a higher age was observed in patients with low BMD compared with patients with normal BMD (*p* < 0.001). The majority of epidemiological and clinical characteristics related to RA were similar in both groups, except for more deteriorated functioning in the group with low BMD compared with that with normal BMD (*p* = 0.003) and lower BMI (*p* = 0.002). In relation to ACPAs, anti-CCP2 antibodies were determined only in 112 and anti-MCV only in 109 patients. Higher titers of anti-MCV were observed in patients with low BMD in comparison with patients with normal BMD (*p* < 0.0001). Instead, there were no differences observed in anti-CCP2 titers (*p* = 0.37). No differences were observed in currently administered doses of corticosteroids between these two groups (*p* = 0.23). The type of conventional synthetic DMARDs (cs-DMARDs), as well as the frequency of using the combination of cs-DMARDs (>1 cs-DMARD prescribed at the same time) had no statistical differences. The second comparison depicted in this table was among the following three subgroups of patients: (a) RA + OP (*n* = 82), (b) RA + osteopenia (*n* = 49), and (c) RA with normal BMD (*n* = 45). Utilizing ANOVA analysis, we observed that the group with normal BMD had lower age (*p* < 0.0001) and higher BMI (*p* = 0.0001). A trend toward lower anti-MCV titers was observed in patients with normal BMD, although this trend did not achieve statistical significance (*p* = 0.06). Moreover, higher OPG titers were observed in OP compared with osteopenia (*p* = 0.03), but not compared with normal BMD.  Table 2 presents a comparison of clinical characteristics between RA patients with the GG genotype versus patients with the GC or CC genotype. No statistical differences were observed between GG genotype carriers versus GC or CC carriers in the majority of the clinical variables. GC or CC carriers had higher anti-MCV titers (*p* = 0.04) and a trend toward higher corticosteroid doses (*p* = 0.09). GG genotype carriers had similar OPG levels compared with GC or CC genotype carriers (111.3 versus 99.7 pg/mL; *p* = 0.32).

Table 3 compares the genotype frequencies between both RA groups that were similar in terms of low BMD and normal BMD (*p* = 0.62). Polymorphic genotype CC was observed in a low frequency in both groups (group 1, low BMD: 3%; group 2, normal BMD: 2.2%), whereas, as expected, GG was the most frequent genotype in both groups (71 and 64%, resp.). Also, Table 3 shows the OR and their 95% CI of the comparison between GG genotype carriers and those of other genotypes and dominant and recessive models, as well as the comparison of allele frequencies between patients with RA with low BMD and patients with RA with normal BMD. No differences in the risk of low BMD were observed in any of these comparisons.

Table 4 shows the risk factors associated with low BMD, in which it is noted that age is a risk (OR = 1.105; 95% CI = 1.041–1.173; *p* = 0.001). Also, positive ACPAS (anti-CCP-positive or anti-MCV-positive) are a risk for low BMD (OR = 3.755; 95% CI = 1.299–10.852; *p* = 0.015). We do not observe an association for risk of low BMD with the presence of any genotype of the *TNFRSF11B* gene rs2073618 polymorphism.

## 4. Discussion

In this study, we observed that the rs2073618 polymorphism of the *TNFRSF11B* gene does not confer a higher risk of low BMD. GG was the genotype most frequently observed in RA independent of the group. Previous studies have been evaluated in postmenopausal women without rheumatic diseases, observing a probable association between polymorphisms in the *TNFRSF11B* gene and OP in different populations, with nonconclusive results. Some of these studies found an association of polymorphisms in the *TNFRSF11B* gene with osteoporotic changes [14, 15, 18–23], whereas other studies did not observe any association of this polymorphism with OP in postmenopausal women [24–26].

To date, to the best of our knowledge, this is the first study to observe the lack of association between this polymorphism and abnormalities in BMD in RA.

Xu et al., on investigating the association between rs2073618 and the presence of RA, found no significant association with an increase in the risk of RA in Chinese Han population [27]. Assmann et al. and Ye et al., in two separate studies, similarly reported no association between rs2073618 and the risk for RA in Caucasian and Chinese population, respectively [28, 29].

Our hypothesis regarding a possible role of *TNFRSF11B* in an increase in the frequency of OP in RA was based on evidence that OPG levels are related to a decrease in OP and that genetic factors producing conformational changes in serum levels might lead to an increase in the loss of BMD. Xu et al., for example, observed that patients with RA have lower serum OPG levels compared with controls [27]. Zhao et al. described that genetic differences in OPG expression can be important for the regulation of bone remodeling in postmenopausal women [12]. In this latter study, Zhao et al. observed a trend toward lower serum concentrations of OPG in GG genotype carriers compared with CC genotype carriers, although this trend failed to achieve statistical significance.

Although the present study is not related to genotypes for risk for low BMD, including OP, we obtained information about other factors related to low BMD. The presence of positive ACPAs in patients with RA could be an important factor. Guler et al. observed that the presence of high anti-CCP titers had an association with low BMD [30]. However, data have not, to our knowledge, been reported on the role of anti-MCV in BMD in patients with RA.

Our exploratory study had several limitations that must be taken into account. This study might reflect only the genetic characteristics of patients with RA from Western population of Mexico. Therefore, a multicenter study including patients from other regions of Mexico should be considered. On the other hand, we consider that although these multicenter studies are required, they probably would not modify our main conclusion that this polymorphism does not have a significant influence on BMD in RA.

In conclusion, the rs2073618 polymorphism of the *TNFRSF11B* gene does not confer a risk for lower BMD in RA. Instead, factors associated with low BMD, including older age, BMI, and deteriorated functioning constituted the main factors for low BMD in patients with RA. However, given the relevance of genetic factors for the development of OP and low BMD in these patients, the search for other polymorphisms explaining the higher prevalence of low BMD in RA compared with other diseases continues to be ongoing.

## Supplementary Material

Supplementary data. Comparison of genotypic and allelic frequencies between subgroups of rheumatoid arthritis with osteoporosis, osteopenia and normal BMD.

## Figures and Tables

**Table 1:** Comparison of selected patient characteristics between rheumatoid arthritis (RA) with low bone mineral density (BMD) versus RA with normal BMD and among subgroups of patients with RA with osteoporosis (OP), osteopenia, and normal BMD.

	Low BMD (*n* = 131)	Normal BMD (*n* = 45)	*p*	Osteoporosis (*n* = 82)	Osteopenia (*n* = 49)	Normal BMD (*n* = 45)	*p*
*Sociodemographic characteristics*							
Age (yr), mean ± SD	62 ± 9	54 ± 7	<**0.0001**	62 ± 8	61 ± 10	54 ± 7^∗∗^^∗∗∗^	<**0.0001**
BMI (kg/m^2^), mean ± SD	27.5 ± 4.1	29.8 ± 4.3	**0.002**	26.9 ± 3.9	28.5 ± 4.1	29.8 ± 4.3^∗∗^	**0.001**
*Disease characteristics*							
Disease duration (yr), mean ± SD	14 ± 10	13 ± 11	0.58	13 ± 10	14 ± 9	13 ± 10	0.75
DAS28, mean ± SD	3.5 ± 1.5	3.4 ± 1.4	0.58	3.7 ± 1.5	3.8 ± 1.5	3.4 ± 1.4	0.37
HAQ-Di score, mean ± SD	0.62 ± 0.60	0.38 ± 0.36	**0.003**	0.49 ± 0.52	0.60 ± 0.71	0.38 ± 0.36	0.11
ESR (mm/hr), mean ± SD	25 ± 10	23 ± 13	0.39	28 ± 12	24 ± 10	24 ± 13	0.18
CRP (mg/mL), mean ± SD	18.6 ± 32.6	12.6 ± 11.8	0.29	21.9 ± 39.6	13.0 ± 14.0	12.6 ± 11.8	0.22
RF (UI/mL), mean ± SD	192 ± 533	74 ± 118	0.24	177 ± 620	213 ± 378	74 ± 118	0.48
ACPAS (+), *n* = 115 (%)	74/90 (82.2)	15/25 (60.0)	0.03	47/56 (83.9)	27/34 (79.4)	15/25 (60.0)	0.06
Anti-CCP2 (RU/mL), mean ± SD	107 ± 126	82 ± 106	0.37	106 ± 120	108 ± 136	82 ± 106	0.67
Anti-CCP2 (+), *n* (%)	58/87 (66.7)	13/25 (52)	0.24	39/55 (70.9)	19/32 (59.4)	13/25 (52.0)	0.23
Anti-MCV (U/mL), mean ± SD	280 ± 363	82 ± 164	<**0.0001**	271 ± 343	295 ± 399	82 ± 164	0.06
Anti-MCV (+), *n* (%)	59/88 (67.0)	7/21 (33.3)	**0.005**	37/55 (67.3)	22/33 (66.7)	7/21 (33.3)	**0.02**
OPG serum levels (pg/mL), mean ± SD	104.0 ± 73.9	118.4 ± 61.1	0.24	115.0 ± 84.5^∗^	85.6 ± 46.9	118.4 ± 61.1	**0.03**
*Treatment characteristics*							
cs-DMARD use, *n* (%)	106 (80.9)	36 (80.0)	1.00	66 (80.5)	40 (81.6)	36 (80.0)	0.66
Monotherapy, *n* (%)	35 (26.7)	16 (35.6)	0.47	24 (29.3)	11 (22.4)	16 (35.6)	0.66
Combinated therapy, *n* (%)	71 (54.2)	20 (44.4)		42 (51.2)	29 (59.2)	20 (44.4)	
Methotrexate, *n* (%)	72 (55.0)	23 (51.1)	0.73	44 (53.7)	30 (61.2)	23 (51.1)	0.57
Leflunomide, *n* (%)	43 (32.8)	13 (28.9)	0.71	24 (29.3)	19 (38.8)	13 (28.9)	0.51
Sulfasalazine, *n* (%)	38 (29.0)	9 (20.0)	0.25	26 (31.7)	12 (24.5)	9 (20.0)	0.35
Azathioprine, *n* (%)	20 (15.3)	7 (15.6)	1.00	13 (15.9)	7 (14.3)	7 (15.6)	1.00
Chloroquine, *n* (%)	17 (13.0)	8 (17.8)	0.46	12 (14.6)	5 (10.2)	8 (17.8)	0.57
Glucocorticoid use, *n* (%)	109 (83.2)	35 (77.8)	0.50	57 (69.5)	35 (71.4)	33 (73.3)	0.91
Glucocorticoid dose (mg), mean ± SD	6.6 ± 8.7	4.8 ± 3.3	0.23	4.7 ± 6.1	6.4 ± 10.2	4.8 ± 3.3	0.37

BMD: bone mineral density; anti-CCP2: 2nd-generation antibodies against citrullinated proteins; anti-MCV: antimutated citrullinated vimentin antibodies; ACPAs: antibodies against cyclic citrullinated peptides/proteins including anti-CCP2 (+) or anti-MCV (+); cs-DMARD: conventional synthetic disease-modifying antirheumatic drugs; DAS28: Disease Activity Score for 28 joints; HAQ-DI: Health Assessment Questionnaire-disability index; ESR: erythrocyte sedimentation rate; CRP: C-reactive protein; RF: rheumatoid factor. Anti-CCP2 (+) was defined as >5 relative units (RU/mL); anti-MCV (+) was defined as >20 RU/mL; combined therapy was defined as the use of two or more cs-DMARDs. Qualitative variables were expressed in frequencies (%) and quantitative variables in means ± standard deviations (SD). Statistical tests: the chi-square test (or the Fisher exact test if applicable) was conducted for comparisons between proportions and independent sample Student *t*-tests were conducted for comparisons between means, and *p* values were obtained comparing low BMD versus normal BMD. Low BMD (including osteopenia or osteoporosis) (*T*-score ≤ −1 SD). Comparisons between differences in means were performed using one-way ANOVA. Dunnett correction was used as the post hoc test in case of statistical significance in the ANOVA. *p* values were obtained comparing OP (*T*-score ≤ −2.5 SD), osteopenia (*T*-score between −1.0 and −2.5 SD), and normal BMD (*T*-score ≥ −1 SD). ^∗^Statistical significance between OP and osteopenia groups (*p* < 0.05); ^∗∗^statistical significance between normal BMD and OP groups (*p* < 0.05); ^∗∗∗^statistical significance between normal BMD versus osteopenia groups (*p* < 0.05).

**Table 2:** Comparison of sociodemographic, clinical, and laboratory characteristics between GG genotype carriers and GC or CC genotype carriers in patients with rheumatoid arthritis (RA).

	GG (*n* = 122)	GC or CC (*n* = 54)	*p*
*Sociodemographic characteristics*			
Age (yr), mean ± SD	60 ± 9	58 ± 9	0.16
Body mass index (kg/m^2^), mean ± SD	28.1 ± 4.4	28.1 ± 4.0	0.95
*Disease characteristics*			
Disease duration (yr), mean ± SD	14 ± 11	13 ± 8	0.70
DAS28 score, mean ± SD	3.5 ± 1.4	3.6 ± 1.6	0.85
HAQ-Di score, mean ± SD	0.49 ± 0.57	0.47 ± 0.52	0.81
Lumbar spine L1–L4 BMD (g/cm^2^), mean ± SD	0.97 ± 0.17	1.00 ± 0.19	0.37
Femoral neck BMD (g/cm^2^), mean ± SD	0.85 ± 0.17	0.86 ± 0.15	0.55
ESR (mm/hr), mean ± SD	25 ± 11	25 ± 11	0.86
CRP (mg/mL), mean ± SD	15.7 ± 25.9	19.1 ± 32.5	0.56
RF (UI/mL), mean ± SD	170.6 ± 524.8	129.7 ± 229.4	0.68
ACPAs (+), *n* = 115 (%)	63/82 (76.8)	26/33 (78.8)	1.00
Anti-CCP2 (RU/mL), mean ± SD	105 ± 131	92 ± 97	0.56
Anti-CCP2 (+), *n* (%)	48/79 (60.8)	23/33 (69.7)	0.40
Anti-MCV (U/mL), mean ± SD	201 ± 309	345 ± 403	**0.04**
Anti-MCV (+), *n* (%)	46/78 (59.0)	20/31 (64.5)	0.67
OPG serum levels (pg/mL), mean ± SD	111.3 ± 71.9	99.7 ± 68.9	0.32
*Treatment characteristics*			
cs-DMARD use, *n* (%)	98 (80.3)	44 (81.5)	1.00
Monotherapy, *n* (%)	36 (29.5)	15 (27.8)	0.93
Combinated therapy, *n* (%)	62 (50.8)	29 (53.7)	0.93
Methotrexate	66 (54.1)	31 (57.4)	0.74
Leflunomide	37 (30.3)	19 (35.2)	0.60
Sulfasalazine	33 (27.0)	14 (25.9)	1.00
Azathioprine	16 (13.1)	11 (20.4)	0.26
Chloroquine	16 (13.1)	9 (16.7)	0.64
Glucocorticoid use, *n* (%)	85 (69.7)	40 (74.1)	0.59
Glucocorticoid dose (mg), mean ± SD	4.54 ± 5.98	6.54 ± 9.83	0.09

RA: rheumatoid arthritis; BMD: bone mineral density; GG: homozygote genotype; GC: heterozygote genotype; CC: polymorphic homozygote genotype; anti-CCP2: 2nd-generation antibodies against citrullinated proteins; anti-MCV: antimutated citrullinated vimentin antibodies; ACPA: antibodies against cyclic citrullinated peptides/protein including anti-CCP2 (+) or anti-MCV (+); cs-DMARD: conventional synthetic disease-modifying antirheumatic drugs; DAS28: Disease Activity Score for 28 joints; HAQ-DI: Health Assessment Questionnaire-disability index; ESR: erythrocyte sedimentation rate; CRP: C-reactive protein; RF: rheumatoid factor. Anti-CCP2 (+) was defined as >5 RU/mL; anti-MCV (+) was defined as >20 RU/mL; combined therapy was defined as the use of two or more cs-DMARDs. Qualitative variables were expressed in frequencies (%) and quantitative variables in means ± standard deviations (SD). Statistical tests: the chi-square test (or the Fisher exact test if applicable) was conducted for comparisons between proportions and independent sample Student *t*-tests were performed for comparisons between differences in means. *p* values were obtained comparing the GG genotype versus GC or CC genotype.

**Table 3:** Evaluation of the *rs2073618* polymorphism as a predictor of low bone mineral density (BMD) in patients with rheumatoid arthritis (RA).

Rheumatoid arthritis (*n* = 176)	Low BMD (*n* = 131)	Normal BMD (*n* = 45)	OR	95% CI	*p*
*Genotype*					
GG, *n* = 122 (%)	93 (71.0)	29 (64.4)	—	—	
GC, *n* = 49 (%)	34 (26.0)	15 (33.3)	—	—	0.62
CC, *n* = 5 (%)	4 (3.0)	1 (2.2)	—	—	
GG versus GC (as a referent)	—	—	1.41	0.68 to 2.95	0.18
GG versus CC (as a referent)	—	—	0.80	0.09 to 7.46	0.46
GC versus CC (as a referent)	—	—	0.57	0.02 to 5.00	0.35
GC versus GG (as a referent)	—	—	0.71	0.34 to 1.48	0.18
CC versus GG (as a referent)	—	—	1.25	0.03 to 11.6	0.46
CC versus GC (as a referent)	—	—	1.76	0.18 to 17.1	0.34
*Genetic models*					
Dominant model (GG versus CC + GC)	—	—	1.35	0.66 to 2.77	0.21
Recessive model (GG + GC versus CC)	—	—	0.72	0.07 to 6.63	0.42
*Alleles, 2n* = 352	*2n* = 262	*2n* = 90			
G allele, *2n* = 293 (%)	220 (84.0)	73 (81.1)	1.22	0.65 to 2.27	0.26
C allele, *2n* = 59 (%)	42 (16.0)	17 (18.9)	0.82	0.44 to 1.53	0.26

RA: rheumatoid arthritis; BMD: bone mineral density; GG: homozygote genotype; GC: heterozygote genotype; CC: polymorphic homozygote genotype; OR: odds ratio risk; 95% CI: 95% confidence interval. *p* values were obtained comparing low BMD versus normal BMD. Low BMD was defined as osteopenia or osteoporosis (OP) (*T*-score ≤ 1 SD).

**Table 4:** Factors associated with low bone mineral density in the logistic regression.

Variables	OR	95% CI	*p*
Age, years	1.105	1.041–1.173	0.001
ACPAS, positives	3.755	1.299–10.852	0.015
BMI, kg/m^2^	—	Not in the model	—
HAQ-DI, score	—	Not in the model	—
Glucocorticoid dose, mg/day	—	Not in the model	—
Genotype (GG + GC + CC)	—	Not in the model	—

ACPAS: antibodies against cyclic citrullinated peptides/proteins, including anti-CCP2 (+) or anti-MCV (+); HAQ-DI: Health Assessment Questionnaire-disability index; multivariate analysis: logistic regression analysis; dependent variable: low bone mineral density (low BMD). Using a stepwise method.
