# Supplementary material for: Polymorphism rs2073618 of the TNFRSF11B (OPG) Gene and Bone Mineral Density in Mexican Women with Rheumatoid Arthritis
Source: J Immunol Res. 2017 Jul 5;2017:7680434. doi: 10.1155/2017/7680434 (PMC5516719; doi:10.1155/2017/7680434)
Supplement: Supplementary file 1 — Supplementary data. Comparison of genotypic and allelic frequencies between subgroups of rheumatoid arthritis with osteoporosis, osteopenia and normal BMD. [file 7680434.f1.docx]

**Supplementary data.** Comparison of genotypic and allelic frequencies between subgroups of rheumatoid arthritis with osteoporosis, osteopenia and normal BMD.

|  | **Osteoporosis**  ***n* = 82** | **Osteopenia**  ***n* = 49** | **Normal**  ***n* = 45** | ***p*** |
| --- | --- | --- | --- | --- |
| *Genotype* $n=176$ |  |  |  |  |
| GG, $n=122 (\%)$*n* = 122 (%) | 58 (70.7) | 35 (71.4) | 29 (64.4) |  |
| GC, $n=49 (\%)$*n* = 49 (%) | 21 (25.6) | 13 (26.5) | 15 (33.3) | 0.87 |
| CC, $n=5 (\%)$*n* = 5 (%) | 3 (3.7) | 1 (2.0) | 1 (2.2) |  |
| *Alleles,* $2n=352$*2n* = 352 | *2n* = 164 | *2n* = 98 | *2n* = 90 |  |
| G allele, $2n=293 (\%)$ *2n* = 293 (%) | 137 (83.5) | 83 (84.7) | 73 (81.1) | 0.51 |
| C allele, $2n=59 (\%)$*2n* = 59 (%) | 27 (16.5) | 15 (15.3) | 17 (18.9) |  |
| GG: homozygote genotype; GC: heterozygote genotype; CC: polymorphic homozygote genotype. *p* values were obtained comparing osteoporosis (T-score ≤ -2.5 ), osteopenia (T-score between -1.0 and -2.5) and normal BMD (T-score ≥ -1). Statistical tests: Chi-square test (or Fisher exact test if applicable) for comparisons between proportions. | | | | |
